# ​​Delayed Diagnosis of Adult Granulosa Cell Tumor: Emphasizing the Importance of Comprehensive Ovarian Mass Evaluation and Standardized Laboratory Reporting

**DOI:** 10.7759/cureus.83069

**Published:** 2025-04-27

**Authors:** Alexandria L Betit, Kavya Penmethsa, Ricky Patel, DO

**Affiliations:** 1 Obstetrics and Gynecology, Regional Medical Center, Anniston, USA

**Keywords:** anti-müllerian hormone, follicle-stimulating hormone, foxl2 mutation, granulosa cell tumor, inhibin, laboratory report formatting, non-epithelial ovarian cancer, post-analytical error, specific molecular diagnosis

## Abstract

Granulosa cell tumors (GCTs) are rare, non-epithelial ovarian tumors that secrete hormones such as estrogen and inhibin. They are classified as either adult GCTs (AGCTs) or juvenile GCTs (JGCTs). Timely detection is essential to control these malignant tumors and minimize the risk of further progression or spread. However, diagnostic challenges exist, as there are no standardized diagnostic criteria or comprehensive laboratory panels for GCT.

In this case, a 36-year-old woman experienced a delayed diagnosis of AGCT, which was incidentally found during a routine ovarian cyst removal. A laboratory formatting error, which placed the quantitative value of inhibin B within the "in range" category, affected the patient’s management course. This highlights the critical need for standardized diagnostic workups and laboratory result formats to improve the accuracy of diagnoses and prevent delays in treatment. Current diagnostic procedures often include imaging and serum markers such as inhibin B and anti-müllerian hormone (AMH). The lack of standardized diagnostic panels and the absence of clear guidance for non-gynecologic oncologists in diagnosing GCTs may contribute to misdiagnosis and delayed referrals. Recommendations include developing a standardized panel for GCTs, incorporating serum markers such as inhibin B, AMH, and follicle-stimulating hormone, and considering adding testosterone or estradiol levels based on clinical presentation. Additionally, molecular testing for a missense mutation in the *FOXL2* gene, which is found in most GCTs, should be incorporated if there is a high clinical suspicion for GCT.

Laboratory report formatting should also be improved to highlight abnormal results clearly, removing confusing "in range" or "out of range" categories and instead focusing on easily recognizable abnormal values. Formatting of result reports should be clear and personalized to the physician and patient, including highlighted reference intervals based on the range closest to the patient’s population. This case exemplifies how standardizing laboratory procedures and diagnostic criteria could lead to better clinical decision-making, timely referrals, and improved patient outcomes in GCT diagnosis and treatment.

## Introduction

Granulosa cell tumors (GCTs) are non-epithelial ovarian neoplasms derived from sex cords and ovarian mesenchyme [1]. They represent 70% of all sex cord stromal tumors but only 5%-8% of all ovarian tumors [1]. While GCTs can occur at any age, they most commonly affect postmenopausal women, with a mean age of diagnosis around 50 years [1,2]. There are two subtypes: juvenile GCTs (JGCTs) and adult GCTs (AGCTs), which are distinguished by their clinicopathologic features [2]. Most cases are adult-type, with AGCTs comprising 95% and JGCTs only 5% of GCTs [2]. AGCTs are more common in peri- and postmenopausal women, JGCTs predominantly occur in prepubertal girls and young women [2].

GCTs typically present as unilateral ovarian masses that secrete estrogen, inhibin, and anti-müllerian hormone (AMH), leading to various symptoms based on subtype [1,2]. Patients may experience abdominal distension, pelvic pain, virilization, and symptoms of hyperestrogenism such as heavy menstruation, mood swings, headaches, sleep disturbances, and breast cysts [3]. This hyperestrogenic state increases susceptibility to conditions such as endometriosis, uterine leiomyomas, gallbladder and thyroid diseases, insulin resistance, and cancers, including endometrial and breast cancer [3].

Currently, diagnostic criteria and laboratory panels for non-epithelial ovarian tumors such as GCTs remain limited. Although the American College of Obstetricians and Gynecologists (ACOG) and the Food and Drug Administration (FDA) endorse some serum laboratory panels, these often lack markers such as inhibin B and AMH [4-6]. Diagnosis is typically confirmed through elevated levels of inhibin A, inhibin B, and AMH, with a combination of AMH and inhibin B offering the highest sensitivity [2]. Imaging via transvaginal ultrasound (TVUS) or computed tomography (CT) often reveals a unilateral solid mass or multilocular cyst [2]. While GCTs are rarely bilateral and are usually confined to the ovary, imaging may detect metastases if suspected [2].

Cytogenetic studies show distinct alterations in GCTs. JGCTs are linked to trisomy of chromosome 22 and deletions in chromosome 6q, often associated with Ollier’s disease and Maffucci's syndrome [2]. *FOXL2* gene mutations are found in over 95% of AGCTs and 10% of JGCTs, serving as a key diagnostic marker [2].

While most GCTs are indolent and detected early, up to 25% can recur after curative surgery, sometimes more than 20 years later [1]. Lymph node metastases are rare, but aggressive GCTs may spread to the omentum, peritoneum, lungs, liver, or brain [1]. This case highlights a 36-year-old female with an incidental finding of AGCT during a routine excision of a presumed benign ovarian mass. The delayed diagnosis resulted from a laboratory quality assurance issue due to improper formatting, emphasizing the need for comprehensive diagnostic criteria and standardized lab panels. Improved guidance from ACOG could benefit non-gynecologic oncologists in the diagnosis and management of adnexal masses, ensuring accurate and timely detection of GCTs and appropriate referrals for treatment.

## Case presentation

A 36-year-old female with a past medical history of venous insufficiency, fibrocystic breast, kidney stones, fibromyalgia, morbid obesity (body mass index >40), hypertension, anxiety, depression, and polycystic ovarian syndrome (PCOS) presented to the obstetrics and gynecology (OBGYN) clinic for a second opinion about a left ovarian mass. She reported the mass was diagnosed four or five years before presentation and was previously attributed to her history of PCOS. The patient has no pertinent family history. She denied tobacco, alcohol, and illicit drug use. Over the course of the year before presentation, the patient began experiencing breast tenderness and swelling, mood changes, weight gain in the hip and waist areas, hair thinning at the scalp, excessive hair growth on her upper lip and chin, fatigue, and worsening low back and pelvic pain. About two weeks before the presentation, the patient developed increased abdominal pain and severe pelvic pain, which radiated to her left leg and affected her ability to walk.

**Figure 1:**
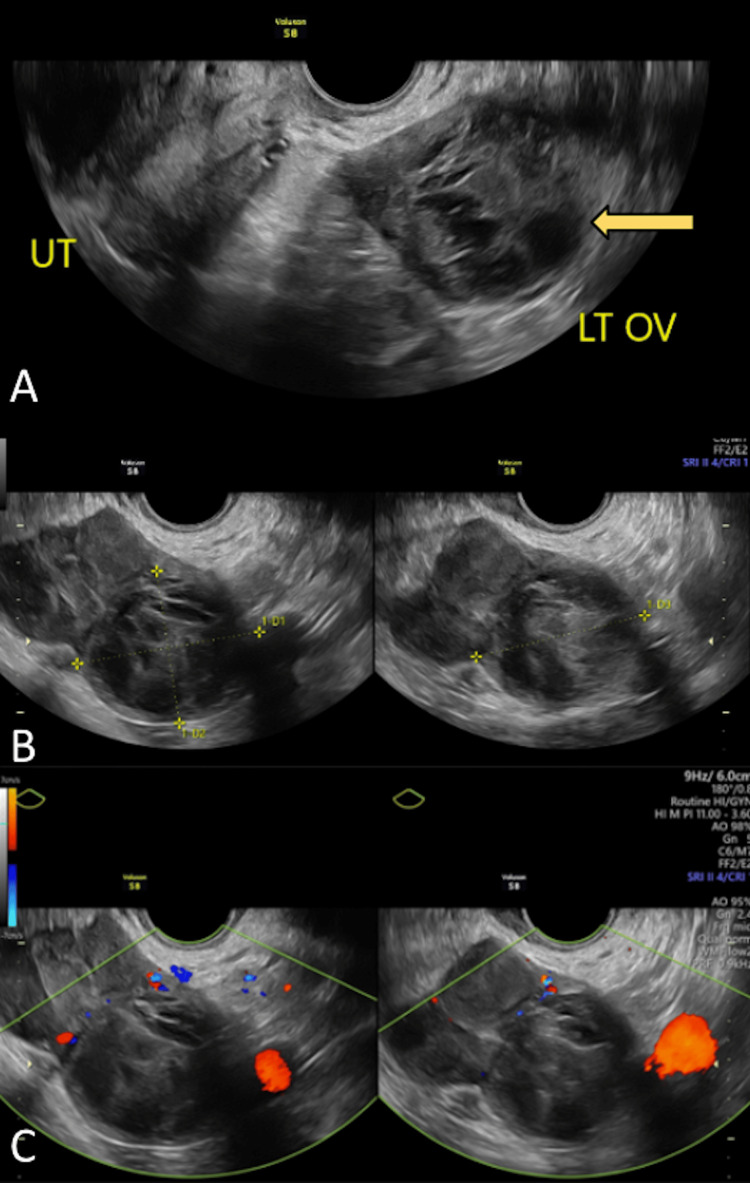
(A-C) Transvaginal Doppler duplex ultrasound of left-sided ovarian mass Figure 1A depicts a gray-scale image of a heterogeneous left ovarian mass with lobulated borders (yellow arrow). Figure 1B demonstrates a gray-scale image of a left ovarian mass measured at 4.4 x 6.7 x 4.5 centimeters (yellow dotted lines). Figure 1C shows a spectral and color Doppler image of the left ovarian mass demonstrating internal blood flow (green outline). LT OV, left ovary; UT, uterus

Upon presentation, the patient reported heavy, irregular menstrual cycles with severe abdominal cramping. She admitted to menstruating every six to seven months, often requiring 10 days of Provera for initiation. On examination, the patient was afebrile, normotensive, and appeared comfortable with an unremarkable abdominal examination. Pelvic examination was remarkable for left adnexal fullness and tenderness. A qualitative urine pregnancy test was negative. A TVUS was performed in the office and showed the left ovary measured 4.4 x 6.7 x 5.5 centimeters with lobulated borders and mixed echo textures that demonstrated internal blood flow (Figures 1A-1C). Given the mixed solid and cystic appearance of the ovarian mass, tumor markers were obtained, including lactate dehydrogenase (LDH), cancer antigen 125 (CA-125), carbohydrate antigen (CA 19-9), carcinoembryonic antigen (CEA), alpha-fetoprotein (AFP), human epididymis protein (HE4), inhibin B, and inhibin A. All tumor markers were reported to be within normal limits except for inhibin B, which resulted at 205 pg/m/L (Table 1). However, the laboratory report was formatted improperly with a disorganized reference range column, so the resulting value was placed within the “in range” column and was not indicated as significant (Table 1). Therefore, upon quick assessment the resulting value was interpreted based on the reference range for males ≥18 years old (Table 1) and was evaluated by the physician as within normal range. Given the low suspicion for malignancy, the need for pathologic diagnosis was discussed and the patient agreed to undergo robotic-assisted laparoscopic left salpingo-oophorectomy.

**Table 1:** Misaligned formatting of the inhibin B laboratory results Resulting value, 205 pg/mL, is depicted above within the “in range” column. Reference ranges are listed above, separated based on age and sex. The female sex is further distinguished based on menopausal status.

INHIBIN B		
	In Range	Out of Range
	205 pg/mL	
Reference Range for Inhibin B		
Age	Male	Female
5-9.9 years	21-166 pg/mL	≤18 pg/mL
10-13.9 years	41-328 pg/mL	≤86 pg/mL
14-17.9 years	54-295 pg/mL	≤123 pg/mL
≥18 years	47-308 pg/mL	
Female	Pre-menopausal	<153 pg/mL
	Post-menopausal	<10 pg/mL

About one month following the initial presentation, robotic-assisted laparoscopic left salpingo-oophorectomy was performed. Upon entering the abdominal cavity, anterior abdominal wall and omental adhesions were discovered surrounding a left-sided ovarian mass. Externally the mass appeared smooth with translucent borders indicating the presence of several solid components. Initially, attempts were made to separate the mass from the ovary given the patient desired future fertility. However, about one to two centimeters into the ovarian cortex away from the border of the mass, yellow appearing solid components were noted inside the ovary. Thus, the pathology department was called, but a frozen section was unavailable at the time of surgery. Therefore, the decision was made to remove the ovary given the abnormal findings.

**Figure 2:**
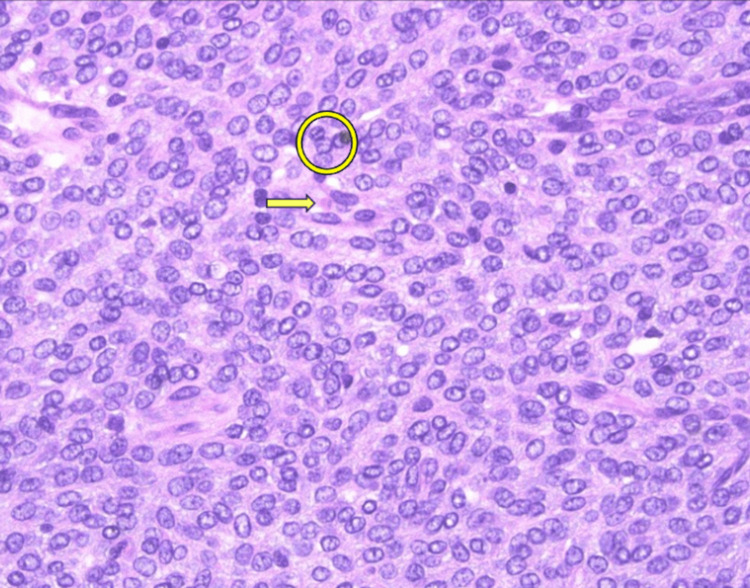
High power (40x) image of AGCT Displays characteristic “coffee bean” morphology due to longitudinal nuclear grooves (yellow circle) and a single Call-Exner body (yellow arrow) in a background of thecal cells. AGCT, adult granulosa cell tumor

Pathology showed an AGCT up to 7.5 centimeters in diameter (Figure 2). Immunoperoxidase stains, including steroidogenic factor (SF-1), calretinin, and inhibin were performed on sections of the tumor. Neoplastic cells were strongly and diffusely positive for SF-1 and calretinin and focally positive for inhibin confirming the diagnosis of AGCT. Upon two-week post-operative follow up, the patient reported that her headaches, breast tenderness, and breast swelling had improved. Given the results of the pathology report, the patient was referred to a gynecologic oncology specialist who proceeded with an endometrial biopsy, which returned negative for atypical endometrial findings. Staging imaging was performed and denoted a clinical stage 1C2 GCT of the ovary. Upon further discussion, the patient stated that she no longer desired future fertility, and robotic-assisted total laparoscopic hysterectomy right salpingo-oophorectomy, resection of right infundibulopelvic ligament, left lymph node dissection, left side wall peritonectomy, bladder peritonectomy, omentectomy, and debulking were conducted. Additional pelvic washings were performed for further pathologic evaluation and showed rare, atypical cells in a background of abundant mesothelial cells leading to a pathologic diagnosis of stage 1C AGCT. Four months following the staging procedure, CT chest/abdomen/pelvis was performed, with no evidence of metastatic disease. The patient reported intermittent numbness and left lower pelvic pain, along with vasomotor symptoms, both consequences of surgical technique and surgical menopause, respectively.

## Discussion

Diagnosis of GCTs should be considered if a patient shows evidence of an adnexal mass and endocrine effects. ACOG created a practice bulletin for the evaluation and management of adnexal masses [4]. Within this practice bulletin, ACOG suggests that the evaluation of adnexal masses should be based on patient characteristics, physical examination findings, imaging results, and serum marker levels [4]. TVUS is the preferred imaging modality used in the evaluation of adnexal masses, while CT is best to evaluate for metastatic disease following TVUS [4]. The bulletin briefly mentions GCTs, indicating solely that these tumors should be suspected in women with solid pelvic masses and irregular or postmenopausal bleeding [4]. However, it does not specify diagnostic criteria or appropriate serum markers for GCT, which are typically not diagnosed using the same laboratory serum markers as more common epithelial ovarian tumors. There is no mention in the practice bulletin that specifically instructs OBGYNs to order different serum tumor markers for patients with high suspicion for GCT. Therefore, further guidance from ACOG on diagnostic criteria and appropriate laboratory serum markers specifically for non-epithelial ovarian cancers may benefit non-gynecologic oncology practitioners in adnexal mass diagnosis and management, ultimately ensuring accurate and timely diagnosis.

Currently, there are no adequate laboratory panels approved by ACOG that are useful in the diagnosis of non-epithelial ovarian tumors such as GCT. ACOG suggests that testing may include serum marker testing in conjunction with imaging to assess the likelihood of malignancy [4]. Current serum laboratory panels used in conjunction with clinical and radiologic evidence supported by the ACOG and the FDA include the multivariate index assay (OVA1 Next Generation) and the risk of ovarian malignancy algorithm (ROMA) [4]. OVA1 Next Generation is a qualitative serum tumor marker panel that includes CA-125 II, transferrin, transthyretin (prealbumin), apolipoprotein A-1, and beta2-microglobulin, and ROMA is an algorithm that includes CA-125, HE4, and menopausal status [4]. However, these panels are based on supportive data from a limited number of patients with non-epithelial ovarian cancer. In the clinical studies testing the OVA1 Next Generation panel, out of 493 patients, only five had non-epithelial ovarian cancer (1%) [5]. In the clinical studies testing the ROMA, out of 461 patients, only two had non-epithelial ovarian cancer (0.4%) [6]. Additionally, these panels are not recommended for the initial evaluation of an adnexal mass, and they exclude serum markers that could assist in diagnosing GCTs. The current use of these panels is primarily to identify patients who would benefit from referral to gynecologic oncologist specialists [4]. These ACOG-recommended laboratory panels exclude inhibin A, inhibin B, and AMH, which are commonly used in the diagnosis of GCTs [2]. Inhibin B and AMH often fluctuate in most healthy premenopausal women, which may limit their utility in AGCT diagnosis [7]. Additionally, almost 15% of GCTs do not produce inhibin B, highlighting that the currently used serum markers do not always correlate with pathology or the extent of disease [7]. However, given the endocrinologic effect of GCTs, we suggest early diagnosis and referral to a gynecologic oncologist with standardized laboratory panels for GCTs, including serum markers inhibin B, AMH, and follicle-stimulating hormone (FSH). A combination of inhibin B and AMH is considered the most sensitive test for GCT diagnosis and recurrence and is reflective of disease activity [2,8]. FSH levels may also be beneficial in diagnosis, given that elevated inhibin levels often result in a reduction of FSH production by the pituitary gland, ultimately resulting in irregular menstrual cycles in premenopausal patients [8]. FSH, inhibin B, and AMH are not included on the OVA1 Next Generation panel, so special consideration should be made toward the creation of an additional separate panel, which includes these serum markers to evaluate for GCTs in cases with a high index of suspicion for GCT. OBGYNs should also consider obtaining total testosterone levels in cases of virilization and estradiol levels for patients with symptoms of hyperestrogenism [2].

Given the limitations of inhibin B and AMH reliability, molecular testing may be a valuable addition. A characteristic missense mutation in the transcription factor *FOXL2*, *FOXL2* 402C>G, is found in 95% of GCTs [7]. This variant has not been seen in other sex-cord stromal tumors or morphological mimics [7]. One experiment found that the use of a specific digital droplet polymerase chain reaction (ddPCR) assay could be used to find the *FOXL2* mutation in circulating tumor deoxyribonucleic acid (ctDNA) in the serum of AGCT patients [9]. The ddPCR was preamplified to target the *FOXL2* mutation with sensitivity and specificity at levels as low as 0.05% [9]. The mutation was detected in the serum of 36% of patients with both primary and recurrent tumors [9]. This established that molecular diagnosis of AGCT can be achieved through non-invasive testing of ctDNA in patients with primary tumors and recurrent disease [9]. In the case of a unilateral ovarian mass, a combination laboratory panel, including inhibin B, AMH, and FSH with additional specific molecular diagnosis should be considered if GCT is clinically suspected.

Gross and microscopic examination is also an important element of GCT diagnosis. Grossly, AGCTs have a variable presentation and can range in size, with an average diameter greater than 10 cm [10]. They are usually solid with mixed cystic components and sections of interspersed yellow-gray fibrous tumor stroma [10]. Histologically, AGCTs present with either high-grade or low-grade differentiated patterns [10]. The key morphological findings of AGCTs are oval, pale cells with “coffee bean grooved” nuclei and Call-Exner bodies [10]. Although the morphology of AGCT is markedly different from other sex-cord stromal tumors, there are some cancers that have been found to have confounding microscopic and immunohistochemical findings [11]. Some endometrioid carcinomas can have sex-cord-like features, mimicking the diffuse pattern of GCTs and having cells with pale nuclei and nuclear grooves, which can be indistinguishable from AGCT in isolation [11]. Immunohistochemistry (IHC) is a tool that can help differentiate AGCTs from common mimics [2]. Classically, GCTs are positive for inhibin, calretinin, and SF-1 [2]. SF-1 and inhibin are known to be the most sensitive for GCTs, but SF-1 is also known to stain positively in all sex-cord stromal tumors, and some GCTs can be completely negative for inhibin on IHC [2]. The use of a FOXL2 stain is also recommended, as it is positive in over 98% of all sex cord stromal tumors and 93% of AGCTs [12]. Overall, the use of SF-1, inhibin, and FOXL2 staining along with proper morphologic correlation should be used for definitive diagnosis of GCT. If morphology is unclear, molecular testing for *FOXL2* should be considered.

The evolution of laboratory medicine, driven by standardized testing, has led to a significant reduction in error rates within the medical field [13]. As laboratories have modernized, the volume of diagnostic reports has increased significantly, outpacing the number of physicians available to interpret them [13]. However, the presentation of laboratory results has not evolved at the same rate, and reporting standards between different laboratories are highly variable with no set guidelines for formatting [13]. Reports are often organized analytically and do not take into account the reader of the report or the patient population from which the results are drawn, which can influence clinical decision-making and patient outcomes [13]. One literature review highlights a need for flexible guidelines that provide clear results by taking into account what information would be important to the physician and which information would be relevant for the specific patient [13]. Most relevant to this case is the formatting of reference intervals (RIs); when a physician is reviewing quantitative results, they are often forced to compare each analyte to its specified RI for several healthy populations [13]. The laboratory often flags results that lie outside the range, but the magnitude of deviation is not taken into account. Sometimes the value of the RI is “within range” but considered abnormal for that specific patient [13]. In this case, the patient’s inhibin B level was not flagged and was considered in range when compared to a normal adult male, but the quantitative value was above the normal RI for premenopausal females. A solution for this specific error would be to highlight the RI of the patient population that the patient is most likely to be a part of within the report.

There has not been enough research to prove that laboratory report formatting significantly impacts clinical decision-making, but there is a growing effort to address post-analytical errors and establish standardized reporting processes [13]. As seen in this case, the formatting of laboratory reports can influence decision-making and patient outcomes. To avoid unnecessary procedures and delays in treatment, laboratory analyses of serum markers should be harmonized and presented in an organized manner. Distinctions such as "in range" and "out of range" should be eliminated to prevent misinterpretation. Instead, we encourage usage of labels such as “H” for high, “L” for low, or “A” for abnormal to highlight results that require further consideration [14]. Harmonizing report formatting could improve patient care, support better physician decision-making, and reduce errors [15,16]. Standardized guidelines for proper formatting should be considered to ensure accurate and effective reporting, as they could have facilitated an earlier referral to a gynecologic oncologist and potentially improved patient care before the initial surgical procedure.

## Conclusions

GCTs are rare, non-epithelial ovarian neoplasms that often present as adnexal masses and are characterized by estrogen and inhibin secretion. These tumors are histopathologically classified into adult and juvenile subtypes, with AGCTs being characterized morphologically by sheets of clear, oval-shaped cells with grooved nuclei and Call-Exner bodies. Despite their generally indolent nature, AGCTs can recur and metastasize, underscoring the need for early detection. However, due to their rarity, standardized diagnostic criteria and laboratory panels for primary diagnosis remain lacking. A recommended diagnostic approach should include serum markers inhibin B, AMH, and FSH, alongside molecular testing for the *FOXL2* mutation. A dedicated GCT panel incorporating inhibin B, AMH, and FSH would improve diagnostic accuracy and facilitate timely referrals to gynecologic oncologists.

In addition to establishing a diagnostic workup, proper analysis of laboratory results is critical to ensuring accurate diagnosis and management of GCT. Laboratory reports should adhere to a consistent formatting standard to reduce variability and minimize confusion. RIs should be clearly highlighted and tailored to relevant patient populations, reducing the risk of misinterpretation. Further research is needed on the impact of laboratory report standardization on clinical outcomes, as well as the development of universal formatting guidelines in laboratory medicine. Proper harmonization of laboratory analyses could prevent unnecessary procedures, expedite appropriate referrals, and ultimately improve patient care in cases of suspected GCTs. Establishing standard formatting guidelines for laboratory results will reduce post-analytic error and assist in timely diagnosis.
